# Compound semiconductor nanotube materials grown and fabricated

**DOI:** 10.1186/1556-276X-6-627

**Published:** 2011-12-12

**Authors:** Likun Ai, Anhuai Xu, Teng Teng, Jiebin Niu, Hao Sun, Ming Qi

**Affiliations:** 1 Key Laboratory of Terahertz Solid-State Technology, Shanghai Institute of Microsystem and Information Technology, Chinese Academy of Sciences, Shanghai, 200050, People's Republic of China; 2 Institute of Microelectronics, Chinese Academy of Sciences, Beijing, 100029, People's Republic of China

**Keywords:** compound semiconductor nanotubes, gas-source molecular beam epitaxy, GaAs/InGaAs/InGaP.

## Abstract

A new GaAs/InGaAs/InGaP compound semiconductor nanotube material structure was designed and fabricated in this work. A thin, InGaAs-strained material layer was designed in the nanotube structure, which can directionally roll up a strained heterostructure through a normal wet etching process. The compound semiconductor nanotube structure was grown by gas-source molecular beam epitaxy. A good crystalline quality of InGaP, InGaAs, and GaAs materials was obtained through optimizing the growth condition. The fabricated GaAs/InGaAs/InGaP semiconductor nanotubes, with a diameter of 300 to 350 nm and a length of 1.8 to 2.0 μm, were achieved through normal device fabrication.

## Introduction

Compound semiconductor nanotubes are a new field that has only caught limited attention. Recently, compound semiconductor nanotubes have been applied in improving existing biological and medical devices and in developing novel devices for gene and drug delivery [1-5]. Traditional technologies fail to produce microtubes with diameters smaller than 10 mm [6]. Previously, a new fabrication method for precise, single-crystal semiconductor micro- and nanotubes was proposed and realized [7,8]. The approach is based on self-rolling of a thin, strained epitaxial heterofilm during its detachment from the substrate in a chemically treated system 'epitaxial heterofilm/sacrificial layer/substrate.' In this technology, the tube diameter can be precisely controlled. This allows feasible large-area assembly and integration with the existing semiconductor technology while maintaining the control of nanotube size and heterojunction formation in the tube wall.

In this work, GaAs/InGaAs/InGaP compound semiconductor nanotube structure materials were designed and grown successfully by gas-source molecular beam epitaxy [GSMBE]. High-quality GaAs, InGaAs, and InGaP epi-layers were obtained through optimizing the growth condition. The fabricated GaAs/InGaAs/InGaP semiconductor nanotubes, with a diameter of 300 to 350 nm and a length of 1.8 to 2.0 μm, were achieved through normal device fabrication. The experimental results indicate that the GaAs/InGaAs/InGaP compound semiconductor nanotubes have a good application prospect.

## Experiments

The GaAs/InGaAs/InGaP compound semiconductor nanotube structure materials with a strained InGaAs layer were grown by a V90 GSMBE system (VG Semicon, East Grinstead, England, UK). The arsenic and phosphorus beams were obtained by thermal cracking of arsine (AsH_3_) and phosphine (PH_3_) at high temperature. Elemental gallium (Ga) and indium (In) were used as group III sources. Silicon (Si) was used as n-type. The GaAs substrates were pre-degassed at about 300°C in the preparation chamber for 30 min to evaporate most of the volatile species, followed by a thermal cleaning process (desorbing) inside the growth chamber. *In situ *reflection high-energy electron diffraction [RHEED] was used to monitor the reconstruction of the substrate surface. The RHEED pattern with continuous strips was observed before and throughout the entire growth process which indicated a good planar surface of the epi-layer. The basic compound semiconductor nanotube structure, as shown in Table 1, consists of a 2,000-nm-thick InGaP layer, a 6-nm-thick In_0.2_Ga_0.8_As strained layer, and a 6-nm-thick GaAs inner wall layer.

**Table 1:** Schematic of the GaAs/InGaAs/InGaP compound semiconductor nanotubes' epitaxial layer structure

Layer	Material	
Inner wall layer	GaAs (*N *= 2E18)	6 nm
Strained layer	In_0.2_Ga_0.8_As	6 nm
Sacrificial layer	InGaP	2,000 nm
	Semi-insulating GaAs substrate	

## Results and discussion

An X'pert high-resolution X-ray diffractometer [XRD] (Philips, Amsterdam, The Netherlands) was used to evaluate the crystalline quality of the epi-layer and its lattice mismatch with the GaAs substrate. Through optimizing the growth condition, high-quality lattice-matched InGaP/GaAs and mismatched In_0.2_Ga_0.8_As/GaAs heterostructures were obtained.

**Figure 1:**
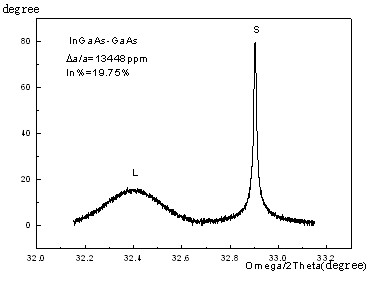
**XRD rocking curve of In_0.2_Ga_0.8_As/GaAs InGaP/GaAs mismatch hetero-epitaxial structure**.

**Figure 2:**
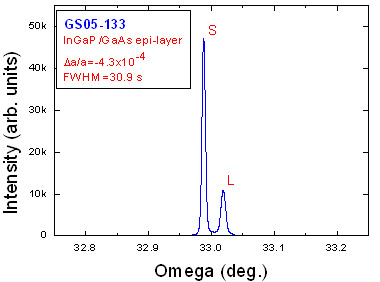
**XRD rocking curve of the epitaxial structure**.

From the XRD rocking curve shown in Figure 1, it was shown that the lattice mismatch between InGaAs and GaAs is 1.3448 × 10^-2 ^and the In content is 19.75%, which illustrate a good crystalline quality and a good composition of the hetero-epi-layer. Figure 2 shows the XRD rocking curves of two InGaP/GaAs epitaxial structures with different compositions. The lattice mismatch is 4.3 × 10^-4^, and the full width at half maximum of the InGaP is 30.9 arc sec. These results illustrate that the InGaAs and InGaP materials have a good crystalline quality.

**Figure 3:**
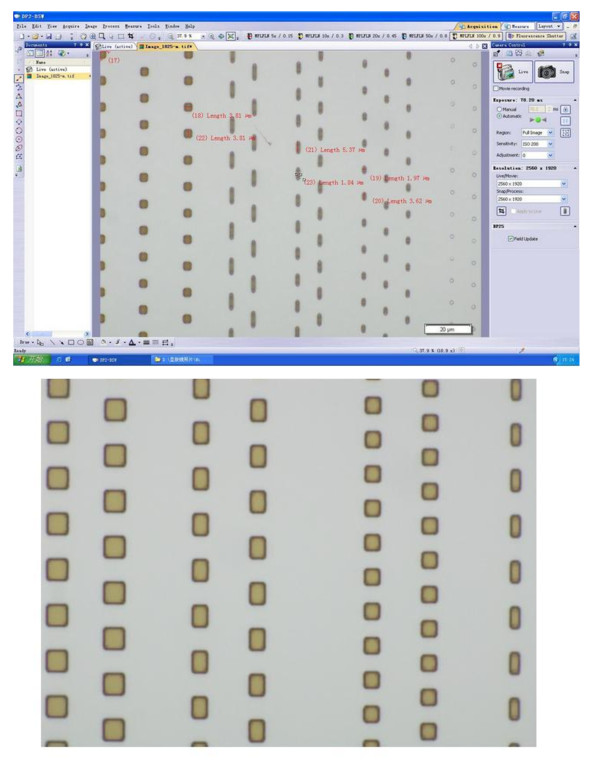
**Microscope image of the photo-etched mask pattern**.

As shown in Figure 3, we designed the photo-etched mask patterns, which have different sizes of 6 × 2 μm, 4 × 4 μm, and 4 × 2 μm in the array patterns. Depending on the different sizes of the array patterns, the compound semiconductor nanotubes were fabricated by photolithography and wet etching process.

The compound semiconductor nanotube structures were fabricated by photolithography and wet etching process. The etching reagent of GaAs, InGaAs, and InGaP materials is shown in Table 2. InGaAs and GaAs materials are etched by citric acid and H_2_O_2 _mixture with a molar ratio of 1:1. InGaP materials are etched by H_3_PO_4 _and HCl mixture with a molar ratio of 3:1. H_3_PO_4 _and HCl etching reagent have a good selective corrosion for etching InGaAs/InGaP materials [9], which can etch InGaP materials, but not InGaAs and GaAs materials. When the InGaP sacrificial layer is etched, the thin, strained InGaAs layer will self-roll and become a tube.

**Table 2:** Etching reagent of GaAs, InGaAs, and InGaP materials

Materials	Etching reagent (molar ratio)
GaAs and InGaAs	Citric acid/H_2_O_2 _= 1:1
InGaP	H_3_PO_4_/HCl = 3:1

**Figure 4:**
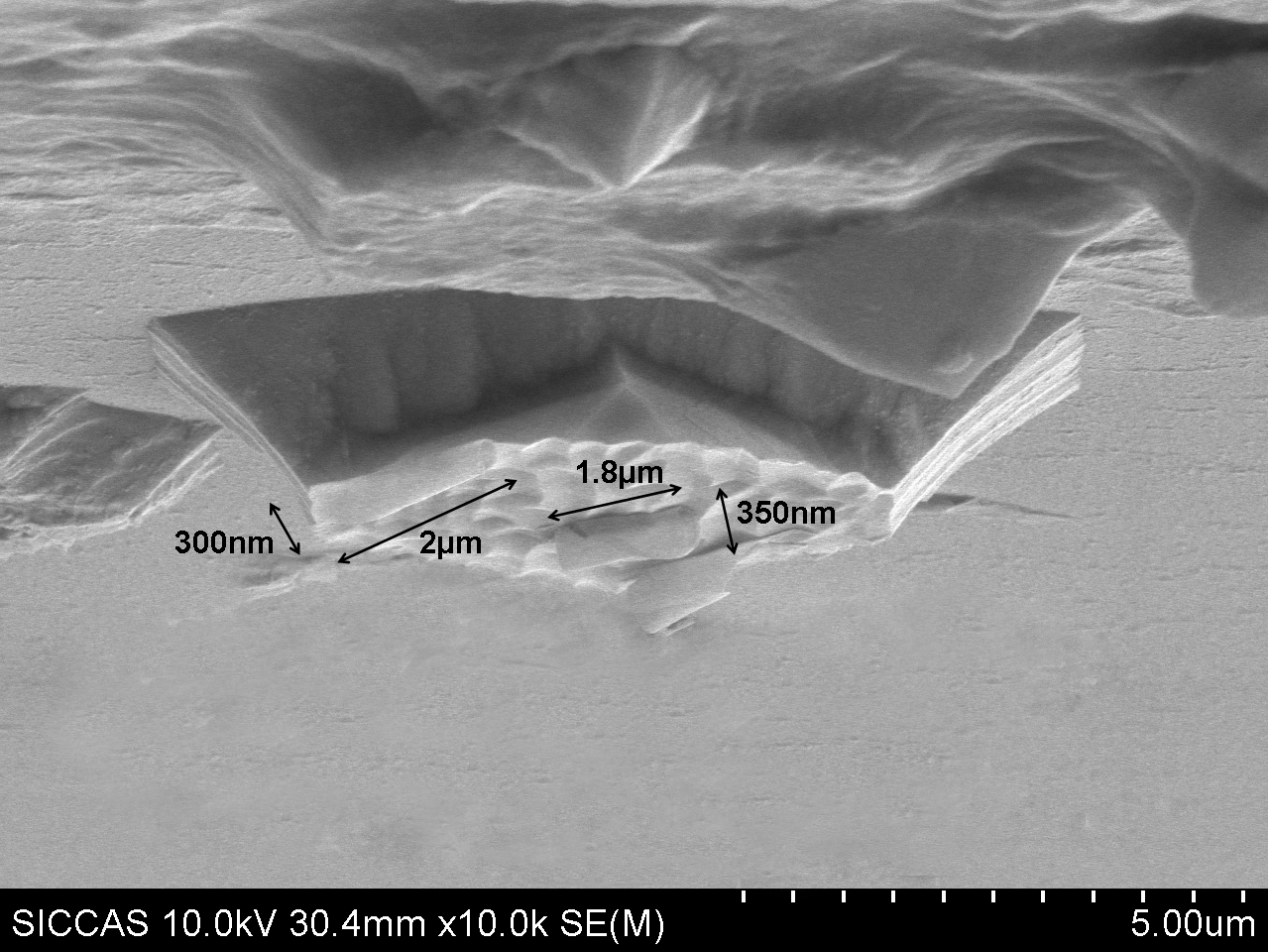
**SEM image of GaAs/InGaAs/InGaP compound semiconductor nanotubes**.

The fabricated GaAs/InGaAs/InGaP semiconductor nanotubes, with a diameter of 300 to 350 nm and a length of 1.8 to 2.0 μm (shown in Figure 4), were achieved through normal device fabrication. The experimental results indicate that the GaAs/InGaAs/InGaP compound semiconductor nanotubes have a good application prospect.

## Conclusions

In summary, GaAs/InGaAs/InGaP compound semiconductor nanotube structure materials were designed and grown successfully by GSMBE. High-quality GaAs, InGaP, and InGaAs epitaxial materials were obtained successfully by optimizing the growth conditions. In the fabrication process, the photo-etched mask patterns were designed and compound semiconductor nanotube structure materials were fabricated by normal photolithography and wet etching process. The compound semiconductor nanotubes with a diameter of 300 to 350 nm and a length of the 1.8 to 2.0 μm were achieved. The experimental results indicate that the GaAs/InGaAs/InGaP compound semiconductor nanotubes have a good application prospect.

## Competing interests

The authors declare that they have no competing interests.

## Authors' contributions

LA, AX, and TT designed and grew the compound semiconductor nanotube materials and participated in the fabrication process. JN carried out the fabrication process. HS participated in the design of the study and performed the statistical analysis. MQ conceived the study and participated in its design and coordination. All authors read and approved the final manuscript.
